# Bariatric Surgery Meaningfully and Durably Improves Long-term Outcomes in Adolescents with Severe Obesity

**DOI:** 10.1016/S2213-8587(16)30315-1

**Published:** 2017-01-06

**Authors:** Thomas H. Inge, Todd M. Jenkins, Stavra A. Xanthakos, John B. Dixon, Stephen R. Daniels, Meg H. Zeller, Michael A. Helmrath

**Affiliations:** Division of Pediatric Surgery (THI, TMJ, LSS, MAH), the Division of Preventative Cardiology (EMU), the Division of Behavioral Medicine (MHZ), the Division of Gastroenterology, Hepatology & Nutrition (SAX), Cincinnati Children’s Hospital Medical Center, Cincinnati, OH, USA; Department of Pediatrics, Children’s Hospital Colorado, Denver, CO, USA (SRD); Clinical Obesity Research, Baker IDI Heart and Diabetes Institute, Melbourne, Australia (JBD)

**Keywords:** bariatric, Roux-en-Y gastric bypass, adolescence, outcome, obesity

## Abstract

**Background:**

Little is known about long-term outcomes of bariatric surgery for severe adolescent obesity, raising questions about durability of early responses to surgery. We therefore analyzed long-term (> 5 years) weight, comorbidity, and safety outcomes of Roux-en-Y gastric bypass (RYGB) in a cohort of young adults who had undergone the operation during adolescence.

**Methods:**

Outcomes of 58 individuals who underwent RYGB for clinically severe obesity at 13–21 years of age were examined. Body mass index (BMI), comorbidities, micronutrient status and other risks were assessed at 5 to 12 years following surgery.

**Findings:**

The cohort had baseline mean (±SD) age of 17± 2 years and median BMI 56 kg/m^2^. At mean follow up of 8 ± 2 years, mean age was 25 years and mean BMI change was −29 ± 14%. Prevalence of elevated blood pressure, dyslipidemia, and type 2 diabetes significantly declined. Mild anemia, hyperparathyroidism, and low vitamin B12 levels were observed in 46% (n=25), 45% (n=22), and 16% (n=8), respectively, at follow-up.

**Interpretation:**

RYGB in adolescence resulted in significant and durable weight reduction and cardiometabolic benefits in young adulthood. Long-term health maintenance following RYGB should focus on adherence to supplements, and screening and management of micronutrient deficiencies.

**Funding:**

This research was supported by investigator initiated grants from Ethicon Endosurgery (grants # 15161 and # 15151·3). The project was also supported by the National Center for Advancing Translational Sciences of the National Institutes of Health, under Award Number UL1TR000077. The content is solely the responsibility of the authors and does not necessarily represent the official views of Ethicon Endosurgery or the NIH.

Severe pediatric obesity, defined as body mass index (BMI) ≥120% of the 95^th^ percentile for age and gender, affects 4.6 million children and adolescents (6.3%) in the U.S.,^1^ threatening health, quality of life, and life expectancy.^2^ Non-operative treatments for severe pediatric obesity--even those of high intensity--have been of limited efficacy.^3^

Bariatric surgery has been used to treat adults with severe obesity for many decades. However for adolescents, access to surgical care has been limited^4^ due in part to lack of long-term evidence of risks, benefits, and durability of weight loss. While we are beginning to understand more about the early and mid-term effects of surgery in youth,^5^ concerns about the late effects of modern bariatric procedures have fueled demand for objective long-term adolescent bariatric outcome data.^6,7^ We hypothesized that clinically significant weight loss and metabolic benefits would be maintained beyond five years following surgery but adverse events related to micronutrient deficiencies would also be observed.

## METHODS

### Study design, recruitment, and enrollment of participants

Seventy four adolescents underwent RYGB surgery during May 2001 to February 2007 at a single pediatric academic medical center. Early outcomes were described previously.^8,9^ Due to aging and transition of clinical care, many were no longer in routine follow-up at this center.^10^ Thus the Follow-up of Adolescent Bariatric Surgery at 5 Plus years (FABS-5+) extension study was designed. FABS-5+ study staff located, recruited, and prospectively conducted a one-time, long-term study visit for each participant.

Inclusion criteria for FABS-5+ consisted of age ≤ 21 years at time of bariatric surgery. Exclusion criteria were inability to complete self-report forms due to developmental delay, or death prior to long-term study visit. However, deaths of any patients who had surgery during this time period were captured. Study procedures were approved by the Cincinnati Children’s Hospital and Medical Center Institutional Review Board. Additional study design details are contained in the webappendix.

**Figure 1:**
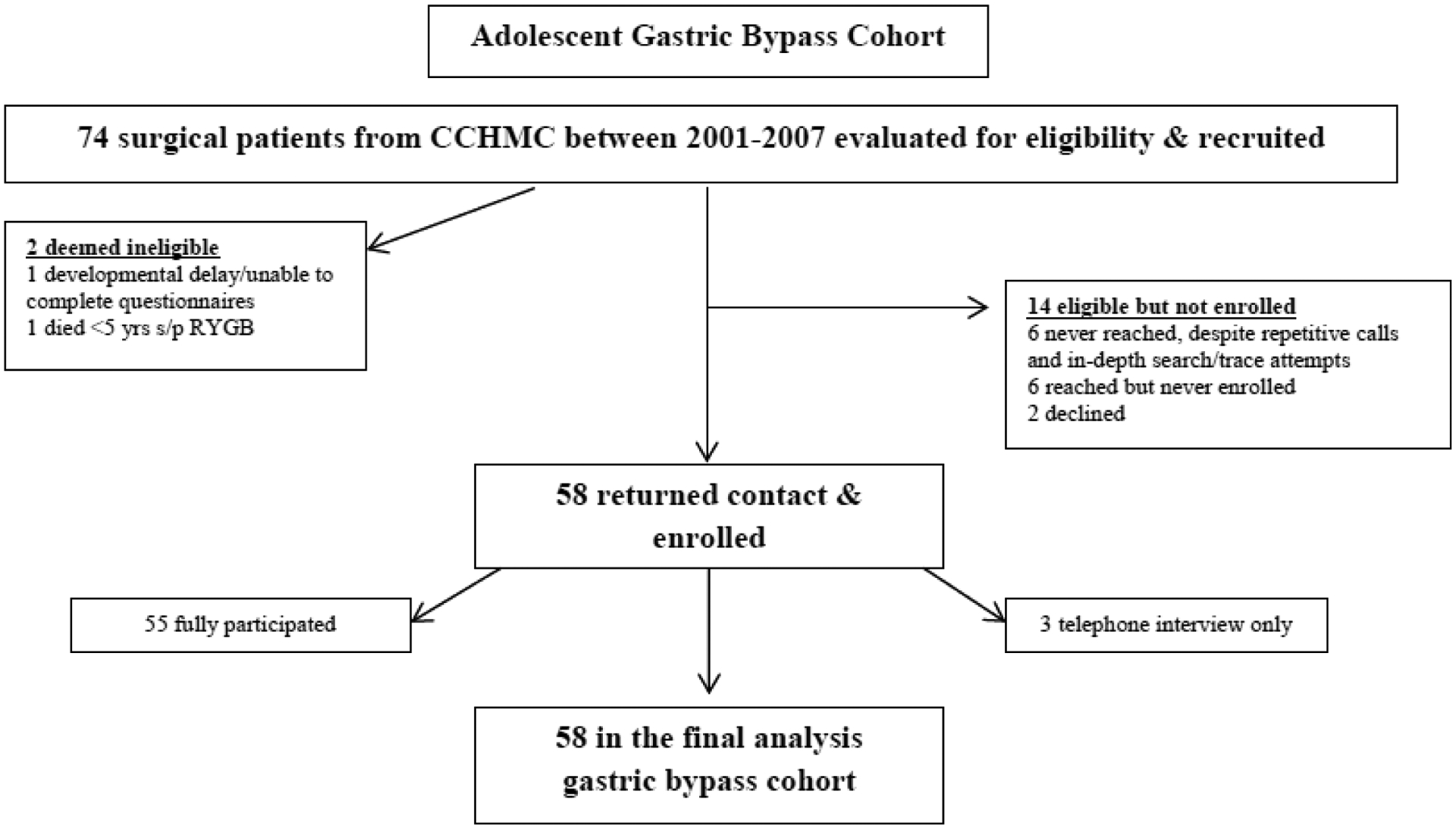
Cohort recruitment

To locate participants, their last known contact information was used, in conjunction with electronic searches including social media and Lexis-Nexis Accurint (http://www.accurint.com). Fifty-eight (80·5% of all eligible) were located and enrolled and data were obtained by direct measure and interview of participants (Figure 1). Baseline characteristics of the 58 participants and 14 other who were eligible but did not participate did not differ significantly by race, age, sex, and BMI (see web appendix). Baseline and one year postoperative data (anthropometric, clinical features, and biochemical measures) were obtained by merging data from prior research databases or by abstraction from clinical records.

Principles of multidisciplinary care and surgical patient selection criteria used in this series have been previously outlined.^11^ The first two individuals in this series underwent RYGB by open laparotomy while all subsequent participants underwent laparoscopic RYGB. All cases were performed by two attending pediatric surgeons using surgical techniques previously described.^9^

### Follow-up study visits and data collection

Long-term follow-up data were gathered by clinical research coordinators either at the medical center or in the participant’s home. For participants who chose to have a home visit, a trained examiner from Examination Management Services, Inc. (Dallas, TX) was dispatched. All data were obtained by direct measurement and a structured health interview. Height was measured to the closest 1·0 mm in standing position. Weight was measured in light clothing to the nearest 0·1 kg on an electronic scale (Tanita model TBF-310, Tokyo, Japan). Blood pressure was obtained using a Welch Allen Spot Vital Monitor 4200B. All blood was collected by phlebotomy and analyzed at our clinical laboratory, with abnormal results communicated back to the participant and/or primary care provider. Each visit lasted approximately four hours and participants were compensated for their time and reasonable travel expenses were reimbursed.

### Data definitions

Detailed descriptions of the research methods used, comorbidity, and remission definitions are provided in the webappendix.

### Statistical analyses

Categorical descriptive measures were presented using frequencies and percentages and continuous variables summarized using means with standard deviations or medians with intra-quartile range. The following outcomes were evaluated using repeated measures linear mixed modeling: height, weight, BMI, fasting insulin, fasting glucose, HbA1c, HOMA-IR, fasting triglycerides, LDL, HDL, systolic blood pressure, diastolic blood pressure. Log transformation of triglycerides, high sensitivity C-reactive protein (hs-CRP), and homeostatic model assessment of insulin resistance (HOMA-IR) was performed to fulfill modeling assumptions. Diabetes, dyslipidemia, and hypertension outcomes were evaluated using repeated measures generalized mixed models. Each model included study visit as the independent predictor term. Estimates of least-squares means and 95% confidence intervals were generated. These models addressed missing data values by means of the maximum likelihood method, under the data missing at random assumption. However, missing data were not considered to meaningfully impact study findings as 96% (222 of 232 visits) of study visits across the four timepoints (baseline, 6 months, 1 year, long-term visit) were completed. Weight values from female participants in their second or third trimester of pregnancy and up to six month postpartum were omitted from the analyses. Baseline and long-term BMI were also evaluated using the Pearson Correlation and simple linear regression. Linear regression was also used to assess the association between BMI at long-term follow-up and the following outcomes: fasting insulin (log), fasting glucose, Hs-CRP (log), HOMA-IR (log), HbA1c, triglycerides (log), LDL, HDL, systolic blood pressure, and diastolic blood pressure. To evaluate the relationship between long-term visit BMI and dyslipidemia and hypertension, risk ratios and predicted probabilities were calculated using Poisson regression with robust variance. Rates for clinical events were calculated as the number of events that occurred, divided by the person-years of observation. Poisson regression with the logarithm of person-years as an off-set parameter was used to calculate unadjusted rates and 95% confidence intervals, expressed per 1000 person-years (i.e., 100 participants followed for ten years). All analyses were conducted using Statistical Analysis Software, v9·4. Reported p-values were two-sided and considered statistically significant when less than 0·05.

## RESULTS

### Cohort characteristics

The analysis cohort was 64% (n=37) female, 86% (n=50) White, and 97% (n=56) non-Hispanic (Table 1). There were no known cases of syndromic or genetic obesity with the exception of one individual with hypothalamic obesity that was due to a prior craniopharyngioma treatment. At the time of bariatric surgery, the mean age was 17 years (range 13–21) and mean BMI was 59 kg/m^2^ (range 41–87 kg/m^2^). Type 2 diabetes, dyslipidemia, and hypertension were observed in 16% (n=9), 86% (n=48), and 47% (n=27) of the cohort, respectively, at baseline (Table 2).

### Educational, employment, and living status at follow-up

At mean follow up of 8.0 ± 1.6 years (range 5.5–12.4 years), the mean age was 25 years (range 20–30). Most (96%, n=51) participants were high school graduates, with 64% (n=34) reporting continuation of their education through college or vocational training (Table 1). Most (66%, n=35) were currently employed part- or full-time or full-time students. The majority (66%, n=35) reported being single, and 49% (n=26) still lived with their parents.

### Weight and BMI change

In the first year following RYGB, an average BMI change of −23±6 kg/m^2^ (39±7% loss) was observed (Figure 2A; Table 3). At long-term follow-up (range 5–12 years), BMI reduction averaged −17±8 kg/m^2^ (−29±14%; p<0·01), corresponding to a mean sustained weight decrease of 50±26 kg (−30±14%). BMI change was also examined by BMI category. All participants had severe obesity (BMI≥40 kg/m^2^) at baseline. At follow-up, 36% (20/55) had a BMI < 35 kg/m^2^; median BMI of this subgroup at baseline and follow-up was 51 kg/m^2^ and 30 kg/m^2^, respectively. One (2%) had achieved normal BMI, while ten (18%) and nine (16%) had achieved overweight (BMI 25 to <30) and class one obese status (BMI 30 to < 35), respectively. However, 64% (35/55) still had BMI ≥35 kg/m^2^ at long-term follow-up, despite significant mean BMI reduction postoperatively. Overall, 87% (48/55) demonstrated ≥10% BMI reduction over the long-term. A strong relationship was observed between baseline BMI and long-term BMI (r=0·75, p<0·01; Figure 2B). Eighteen participants (31%) were 18 years of age or older at the time of surgery and there was no difference in weight loss for those who were greater than compared to those less than 18 years old at baseline. Similarly, there was no relationship detected between age at baseline and BMI change over the long term.

### Cardiometabolic outcomes

Mean fasting insulin values decreased from 38±20 to 8±7 μU/mL (p<0.01), mean glucose from 5.37±0.94 to 4.76±1.97 mmol/l (p=0.055), and mean HOMA-IR from 9±6 to 1.5±1.3 (p<0·01) at follow-up (Table 4). Additionally, accounting for diabetes medication usage and measured HbA1c values in the case definition, the prevalence of type 2 diabetes decreased from 16% (n=9) to 2% (n=1) at long-term follow-up (p=0·03), with diabetes remission in 88% (n=7). No incident cases of diabetes were observed (Table 2).

Fasting median triglyceride levels significantly decreased by 40% from 1.43 to 0.86 mmol/l, (p<0·01), while LDL values decreased by 12% from 2.78±0.68 to 2.44±0.79 mmol/l (p<0·01). HDL cholesterol increased by 60% from 0.91±0.19 to 1.46±0.45 mmol/l (p<0·01). Accounting for both changes in lipid values (considering age-appropriate norms at baseline and follow-up as described in webappendix) and lipid lowering medication use in the case definition, the prevalence of dyslipidemia fell from 86% (n=48) at baseline to 38% (n=21) at follow-up (p<0·01). Remission of baseline dyslipidemia was observed in 64% (n=29), while incident dyslipidemia was noted in four of the eight who did not have dyslipidemia at baseline, due only to HDL levels mildly below adult targets. Three of these incident cases were females with follow-up HDL values in the range of 1.03–1.27 mmol/l, below the adult female target value of 1.29 mmol/l. The remaining incident case was a male with an HDL value of 0.98 mmol/l, just below the male normal HDL target of 1.03 mmol/l.

Mean (±SD) systolic and diastolic blood pressures remained similar over time from 126±13 to 124±15 mmHg (p=0·59) and 74±10 to 73±11 (p=0·82), respectively. Accounting for age-appropriate norms for blood pressure (described in webappendix) and medication use in the case definition, the prevalence of hypertension fell from 47% (n=27) at baseline to 16% (n=9) at follow-up (p<0·01). Remission of hypertension was observed in 76% (n=19) in this group, while incident hypertension was observed in 10% (n=3 of 10 participants without hypertension at baseline; Table 2). Further exploration of these three participants with incident hypertension revealed that all experienced suboptimal BMI change at long-term follow-up (+0·5%, −8%, −0·1%). The participant with the 0·5% increase in BMI met the hypertension definition due to medication use while the other two subjects had elevated systolic and diastolic blood pressure values, respectively.

### Relationship between long-term follow-up BMI and cardiometabolic status

While the cohort on average experienced dramatic improvements in weight and health status over time, at long-term follow-up the majority of participants remained significantly obese with 64% (n=37) having a BMI ≥ 35 kg/m^2^. This finding raises a fundamental question of whether higher residual BMI at long-term follow-up, despite prior significant weight reduction, heightens risk of adverse future health outcomes. Indeed, regression modeling revealed a significant relationship between follow-up BMI and cardiometabolic risk. For every 10 kg/m^2^ increase in follow-up BMI, a 34% greater risk of dyslipidemia was observed (Table S1, Figure S2; p=0·006), while a 46% greater risk of hypertension was noted (Table S1, Figure S2; p=0·01). Finally, each 10 kg/m^2^ rise in BMI at the long-term follow-up visit was accompanied by an increase of 66% in hs-CRP (p=0·002), 25% in insulin (p=0·0004), and 24% in HOMA-IR (p=0·003; Table S2).

### Micronutrient status

Low iron, ferritin, and hemoglobin levels were found in 69% (n=35), 63% (n=32), and 46% (n=25) respectively, at long-term follow-up (Table 5). Of those who had both hemoglobin and ferritin measured, 19 of 23 (83%) with low hemoglobin had concurrent low ferritin, suggestive of iron deficiency anemia in the majority of those with anemia. Vitamin D levels were low (< 20 ng/ml) in 78% (n=39) of the cohort, with elevated parathyroid hormone found in 45% (n=22). Low vitamin B12 levels were present in 16% (n=8) of the cohort. Abnormalities in serum albumin, calcium, alkaline phosphatase, and folate were distinctly uncommon.

### Clinical events

Participants were systematically queried regarding procedures during the postoperative period. Obstetric and gynecologic procedures were the most common procedures, occurring in 46% (n=17) and 19% (n=7) of women, respectively (Table 6). Of the 17 women in this study who became mothers during the postoperative period, 11 gave birth to only one child, four gave birth to two children, and two gave birth to three children. One participant reported giving birth to a pre-term infant and another reported having hypertension related to her pregnancy. Several procedures, transfusion, and infusions were considered probably or possibly related to the prior RYGB procedure. Upper endoscopy, cholecystectomy, repair of gastrointestinal perforation, colonoscopy, and exploratory laparoscopy were observed in 22% (n=13), 21% (n=12), 5% (n=3), 3% (n=2), and 3% (n=2), respectively. Six blood and micronutrient infusions were reported by three participants.

Two deaths occurred in patients who underwent operation within the 2001–2007 period. The first patient developed infectious colitis and died at nine months postoperatively as previously described,^12^ and did not therefore qualify for this long-term analysis. The second participated in this study at six years postoperatively, but died of events unrelated to surgery two years after his study visit (see webappendix).

## DISCUSSION

Participants in this study experienced major and sustained reductions in BMI and significant improvements in cardiometabolic health 5–12 years following RYGB. Despite reduction in BMI, there was a “floor effect” of the intervention. Most participants with the highest BMI values at baseline remained with severe obesity at long-term follow-up, and persistence of health risks were linked to higher postoperative weight status. While some participants reported undergoing additional procedures, most were not related to abdominal or gastrointestinal problems. In addition, several nutritional deficiencies were detected, though generally mild and manageable.

Once present, pediatric obesity, and particularly severe pediatric obesity, is exceedingly difficult to reverse.^13,14^ Very little is known about the long-term BMI and health outcomes of youth with severe obesity, but adolescents in the moderately obese category are far more likely to achieve a normal weight than those with severe obesity following both school-based^15^ and conventional behaviorally-based^16^ interventions. The higher burden of cardiovascular risks and a worse prognosis for weight loss in youth with severe obesity,^17^ has led to consideration of more aggressive, surgical treatment approaches.

Exposure of youth to severe obesity and cardiometabolic risk factors^17–19^ leads to premature progression of cardiovascular disease, limiting both quality and quantity of life.^2^ Type 2 diabetes is also a particularly aggressive condition with rapid progression to need for exogenous insulin^20^ and progression of cardiovascular risk factors and proteinuria over time.^21^ The long-term surgical results of this cohort provide reassurance that the early improvements in weight, dyslipidemia, type 2 diabetes, and elevated blood pressure are in fact durable. These findings, in conjunction with the previously documented survival advantage of bariatric surgery demonstrated in numerous other studies^22^ lead us to speculate that use of surgery in adolescence will translate into longer, healthier, and more productive lives for these individuals. However, even longer-term and controlled study will be needed to formally test this hypothesis.

Despite the very favorable weight and comorbidity responses to surgery, these data also highlight a potential disadvantage of offering surgery late in the course of severe weight gain. Nearly two-thirds of this cohort remained severely obese at long-term follow-up and we found a significant relationship between higher follow-up BMI and numerous cardiometabolic risk factors (dyslipidemia, hypertension, insulin resistance, and inflammation) which may well progress further over time. Optimal management of these individuals will require adherence to diet and lifestyle patterns which promote maintenance of weight loss. In addition, adjunctive medications for management of residual cardiovascular risks and obesity may be needed in some to avoid end organ damage^23^.

These data also thus suggest that the timing of surgery during the accumulation of excess weight in adolescence should be carefully considered. Surgical intervention earlier after the diagnosis of severe obesity (e.g., at BMI 35–40 kg/m^2^) may result in more complete reversal of severe obesity and cardiometabolic risks than when surgery is offered to adolescents who have progressed to the higher BMI values of those enrolled in this current study. Notably, the recommendation for consideration of surgery in adolescents at BMI values of 35–40 kg/m^2^ with other clinical indications is entirely consistent with advice contained in numerous peer-reviewed clinical practice guidelines^24–26^ and National Institutes of Health recommendations^27^ for use of surgery in adolescents. While use of surgery in pre-adolescents is an area of increasing interest, health and safety outcomes in these younger age groups are lacking.

While the health benefits of RYGB for adolescents are apparent, some long-term adverse nutritional effects were also seen in this cohort. Mild iron deficiency anemia was found in nearly half of this surgical cohort, with several receiving blood, iron, or vitamin infusions, further highlighting the importance of regular supplementation and monitoring for deficiency states. Low vitamin D levels were highly prevalent, with nearly half the cohort having elevated PTH. This suggests a negative impact on bone health, but future long-term study including markers of bone turnover and bone density is warranted to further define the prevalence and severity of metabolic bone disease.^28,29^ Post-RYGB recommendations include routine daily supplementation with a multivitamin, vitamin B12, vitamin D, calcium and iron. However, in those with rising parathyroid hormone, additional vitamin D is also required. Since adherence to supplements among adolescents is difficult to achieve,^30^ greater focus on self-management, appropriate nutritional monitoring by primary care providers in the medical home, and research efforts to discern which patients are at greatest risk for micronutrient deficiencies are needed.

Notable strengths of this study include the successful follow-up and enrollment of 80% of all eligible subjects, despite known difficulties in achieving satisfactory follow-up of bariatric cohorts over long periods (particularly problematic for highly mobile adolescent and young adult populations).^10^ In addition, long-term data was prospectively collected, including direct measures of anthropometrics and laboratory values, as well as a structured health interview by trained study staff. There are several limitations of this study. First, the lack of a well-matched non-operative control group similarly exposed to severe pediatric obesity and similarly motivated to undergo an intensive weight loss intervention limits our ability to judge the competing risks of not undergoing surgery. Next, although the proportion of females among adolescents with severe obesity in the U.S. is similar^1^, the current cohort is skewed to female gender, white race and non-Hispanic ethnicity, which hinders outcome assessments for other important demographic subgroups at risk of severe obesity. This issue highlights the need for dedicated efforts to sample underrepresented populations, a goal which may be partially achieved using the power of large networked databases in a recently funded Patient Centered Outcomes Research Initiate (PCORI) collaborative study^31^. In addition, despite the extraordinary measures that were taken to locate and recruit all eligible for this study, nearly 20% of those eligible were not able to participate, suggesting that inclusion bias could be present. However, our analysis demonstrated no significant baseline differences between those who did and did not participate in long-term follow-up, suggesting no systematic baseline bias. Therefore we feel that the inferences made from this sample are representative of the entire surgical cohort of 74 individuals and a valuable addition to our knowledge of long-term outcomes in adolescents after bariatric surgery.

In conclusion, ascertainment of long-term adolescent bariatric outcomes is possible and demonstrates excellent maintenance of weight loss and improved health trajectories overall. However, these benefits were achieved with some attendant risks of micronutrient deficiencies, and requirement for additional gastrointestinal procedures related to surgery, providing important data to inform treatment decisions for families. On balance, these data suggest that bariatric surgery performed in adolescence provides greater long-term benefit than risk. Additional research will be needed to determine whether the health benefits observed will translate into improved life expectancy.

## Supplementary Material

Supplementary Appendix

## Figures and Tables

**Figure 2:**
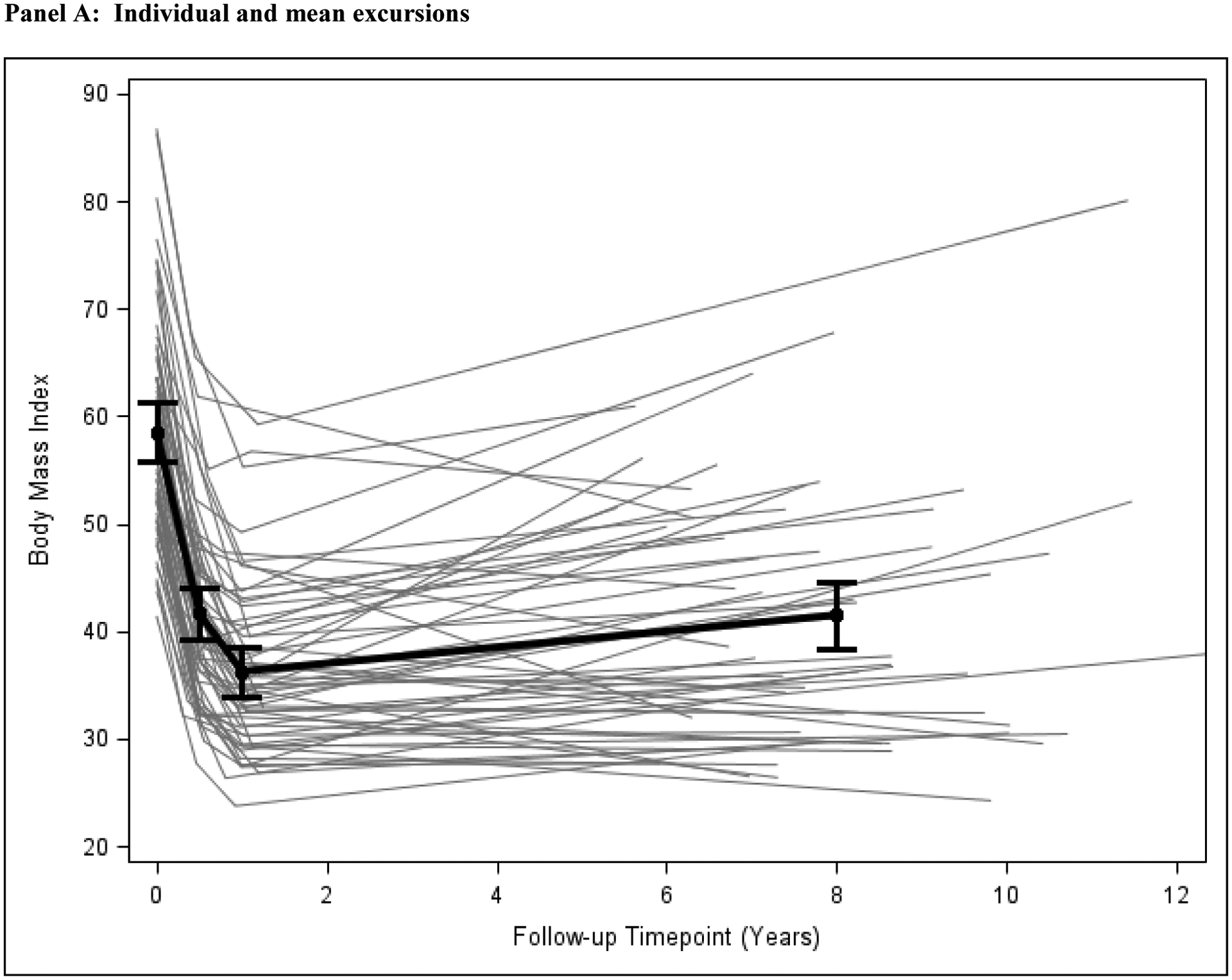
BMI change BMI values for the baseline, 6 month visit, 12 month visit, and the long-term FABS-5 study visit were plotted for each individual (gray lines) and for the mean value (± 95% confidence intervals) of BMI and time for the entire cohort (black thick line).

**Figure 2:**
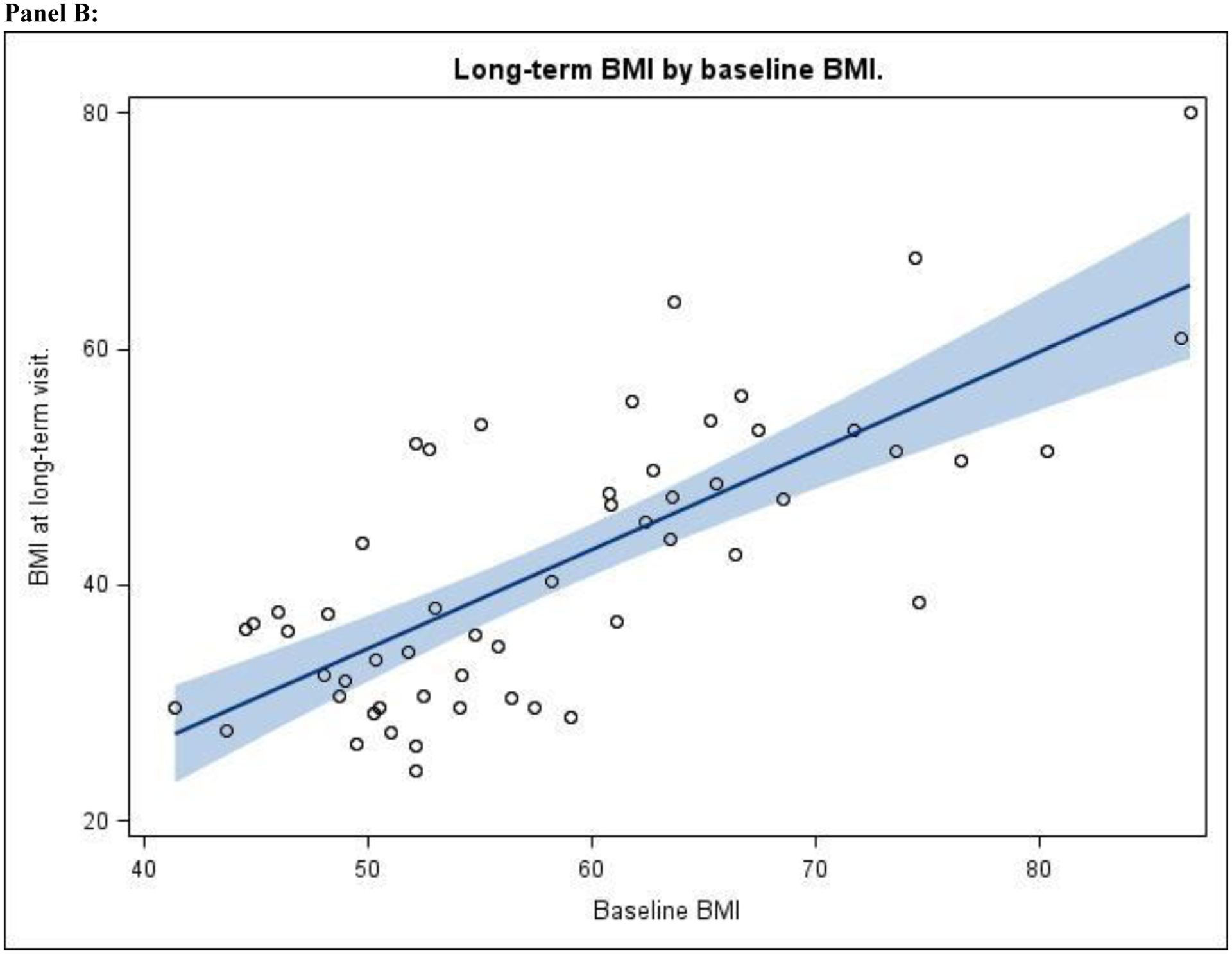
BMI change A scatterplot was created using baseline BMI prior to bariatric surgery (abscissa) and the BMI at the time of the long-term follow-up visit (ordinate) for each participant. The regression line with 95^th^ confidence interval (gray shading) was then plotted using SAS. The Pearson correlation coefficient (r=0.75, p<0·01) was calculated which described the significant association between these two BMI values.

**Table 1.** Surgical Cohort Characteristics.

Characteristic	Mean (SD) or Pct. (n)
Age at surgery (years)	17.1 (1.71) Range: 13.7–21.4
Time since surgery at longitudinal visit (years)	8.0 (1.62) Range: 5.4–12.5
Age at longitudinal visit (years)	25.1 (2.43) Range: 20.7–29.9
	
Sex	
Female	63.8% (37)
Male	36.8% (21)
	
Race	
White	86.2% (50)
Black	12.1% (7)
Multi-race	1.7% (1)
	
Ethnicity	
Hispanic	3.4% (2)
Non-Hispanic	96.6% (56)
	
Baseline Body Mass Index (kg/m^2^)	58.5 (10.46) Range: 41.4–86.8
40–49 kg/m^2^	20.7% (12)
50–59 kg/m^2^	37.9% (22)
60–69 kg/m^2^	27.6% (16)
70 + kg/m^2^	13.8% (8)
	
Marital Status at Longitudinal Visit^a^	
Single	66.0% (35)
Engaged	15.1% (8)
Married	11.3% (6)
Divorced	1.9% (1)
Separated	5.7% (3)
	
Educational Attainment at Longitudinal Visit^a^	
Less than high school	3.8% (2)
High school graduate	32.1% (17)
Some college	54.7% (29)
College graduate	9.4% (5)
	
Employment status at Longitudinal Visit^a^	
Full-time employment	26.4% (14)
Part-time employment	24.5% (13)
Full-time student	15.1% (8)
Unemployed	34.0% (18)
	
Medical Insurance Status at Longitudinal Visit^a^	
Yes	71.7% (38)
No	24.5% (13)
Don’t know	3.8% (2)

a n=6 missing.

**Table 2.** Prevalence, Remission, and Incidence of Comorbid Conditions.

	Baseline		FABS5			
	Prevalence		Prevalence		Remission^b^	Incidence^c^
	Observed no. of participants / total no. (%)	Model-estimated^a^ Percentage (95% CI)	Observed no. of participants / total no. (%)	Model-estimated^a^ Percentage (95% CI)	Observed no. of participants / total no. (%)	Observed Percentage (95% CI)
Diabetes	9/56 (16.1%)^a^	16.1% (8.5,28.3)	1/55 (1.8%)^b^	1.8% (0.2,12.0)	7/8 (87.5%)^c^	0/45 (0%)^a^
Dyslipidemia	48/56 (85.7%)^a^	85.7% (73.8,92.8)	21/55 (38.2%)^b^	38.2% (26.3,51.7)	29/45 (64.4%)^b^	4/8 (50.0%)
Hypertension	27/57 (47.4%)^c^	47.4% (34.7,60.4)	9/55 (16.4%)^b^	16.4% (8.7,28.7)	19/25 (76.0%)^a^	3/29 (10.3%)^c^

a Generalized mixed models were used to calculate the modeled results.

b Remission was calculated as the number of participants (with sufficient data to define comorbidity state) who do not have the condition at long-term visit divided by the number of participants (with sufficient data to define comorbidity state) who had the condition at baseline.

c Incidence was calculated as the number of participants (with sufficient data to define comorbidity state) who have the condition at long-term visit divided by the number of participants (with sufficient data to define comorbidity state) who did not have the condition at baseline.

**Table 3.** Anthropometric Characteristics.

	Baseline		6 months		1 year		Long-term follow-up	
	Observed Mean (SD)	Model-estimated^a^ Mean (95% CI)	Observed^b^ Mean (SD)	Model-estimated^a^ Mean (95% CI)	Observed^c^ Mean (SD)	Model-estimated^a^ Mean (95% CI)	Observed^d^ Mean (SD)	Model-estimated^a^ Mean (95% CI)
Height (cm)	170.6 (9.62)	170.6 (168.0,173.1)	170.1 (10.41)	170.5 (168.0,173.1)	170.9 (9.64)	170.8 (168.3,173.4)	170.3 (9.69)	170.4 (167.8,172.9)
Absolute change from baseline	NA	NA	−0.04 (0.77)	−0.03 (−0.23,0.18)	0.24 (0.85)	0.27 (0.03,0.50)	−0.25 (1.13)	−0.25 (−0.54,0.05)
Percent change from baseline	NA	NA	−0.03% (0.45)	−0.02% (−0.14,0.10)	0.14% (0.49)	0.16% (0.02,0.29)	−0.14% (0.67)	−0.14% (−0.32,0.03)
								
Weight (kg)	170.8 (37.01)	170.8 (161.1,180.6)	121.8 (31.38)	120.9 (112.6,129.2)	106.3 (27.51)	105.4 (98.2,112.7)	122.0 (37.56)	120.9 (111.0,130.9)
Absolute change from baseline	NA	NA	−50.1 (13.73)	−50.1 (−53.8,−46.5)	−66.3 (17.57)	−65.6 (−70.4,−60.9)	−49.9 (25.57)	−50.0 (−56.8,−43.1)
Percent change from baseline	NA	NA	−29.4% (6.41)	−29.4% (−31.1,−27.7)	−38.5% (6.86)	−38.4% (−40.3,−36.5)	−29.3% (13.90)	−29.5% (−33.2,−25.7)
								
Body Mass Index (kg/m^2^)	58.5 (10.46)	58.5 (55.8,61.3)	42.2 (10.92)	41.4 (39.0,43.8)	36.3 (8.02)	36.0 (33.8,38.1)	41.7 (12.02)	41.5 (38.4,44.7)
Absolute change from baseline	NA	NA	−17.1 (4.46)	−17.1 (−18.3,−15.9)	−22.8 (5.50)	−22.6 (−24.1,−21.1)	−16.9 (8.17)	−17.0 (−19.2,−14.8)
Percent change from baseline	NA	NA	−29.3% (6.53)	−29.4% (−31.1,−27.7)	−38.7% (6.69)	−38.6% (−40.5,−36.7)	−29.2% (13.74)	−29.3% (−33.0,−25.6)

a Linear mixed models were used to calculate the modeled results.

b Data are for 56 of 58 subjects.

c Data are for 51 of 58 subjects.

d Data are for 55 of 58 subjects, with 2 subjects excluded due to pregnancy.

**Table 4.** Laboratory values by study visit.

	Baseline	Long-term Follow-Up
**Fasting Insulin (uU/mL), n**	53	48
Observed, mean (SD)	37.8 (20.03)	8.3 (7.30)
Model-estimated^a^, mean (95% CI)	37.6 (32.2,43.1)	7.0 (5.6,8.5)
		
**Fasting Glucose (mmol/L), n**	49	48
Observed, mean (SD)	5.37 (0.94)	4.76 (1.97)
Model-estimated^a^, mean (95% CI)	5.37 (5.11, 5.65)	4.75 (4.17, 5.34)
		
**HbA1c (%), n**	31	50
Observed, mean (SD)	5.3 (0.65)	5.2 (1.30)
Model-estimated^a^, mean (95% CI)	5.3 (5.1.5.6)	5.2 (4.9,5.6)
		
**HOMA-IR, n**	48	45
Observed, mean (SD)	9.0 (5.68)	1.5 (1.25)
Model-estimated^a^, mean (95% CI)	9.2 (7.6,10.9)	1.5 (1.1,1.9)
		
**Fasting Triglycerides (mmol/L), n**	50	45
Observed, Median (Q1,Q3)	1.43 (1.12, 2.05)	0.86 (0.68, 1.39)
Model-estimated^a^, mean (95% CI)	1.45 (1.27, 1.66)	0.99 (0.86, 1.13)
		
**LDL (mmol/L), n**	50	50
Observed, mean (SD)	2.78 (0.68)	2.44 (0.79)
Model-estimated^a^, mean (95% CI)	2.78 (2.59, 2.97)	2.44 (2.22, 2.67)
		
**HDL (mmol/L), n**	50	50
Observed, mean (SD)	0.91 (0.19)	1.46 (0.45)
Model-estimated^a^, mean (95% CI)	0.91 (0.86, 0.96)	1.45 (1.32, 1.58)

a Linear mixed models were used to calculate the modeled results.

**Table 5.** Micronutrients Abnormalities at the Long-term Visit^a^

		Female			Male			Total	
	n	Mean (SD) or Median (Q1,Q3)	Abnormally low, n (%)^b^	n	Mean (SD) or Median (Q1,Q3)	Abnormal, n (%)	n	Mean (SD) or Median (Q1,Q3)	Abnormally low, n (%)^b^
Albumin (g/dL)	35	3.9 (0.38)	1 (2.9%)	17	4.1 (0.43)	0 (0.0%)	52	3.9 (0.41)	1 (1.9%)
Serum Iron (mcg/dL)	35	37.6 (25.22)	27 (77.1%)	16	78.3 (49.40)	8 (50.0%)	51		35 (68.6%)
Ferritin (ng/dL)	35	5.0 (3,10)	23 (65.7%)	16	16.5 (9.5,39.5)	9 (56.3%)	51	8.0 (4.0,15.0)	32 (62.8%)
Hemoglobin (g/dL)	36	11.5 (1.85)	19 (52.8%)	18	13.8 (1.33)	6 (33.3%)	54	12.3 (2.02)	25 (46.3%)
Mean Corpuscular Volume (fL)	35	78.8 (9.60)	19 (54.3%)	18	83.9 (7.15)	4 (22.2%)	53	80.5 (9.10)	23 (43.4%)
Vitamin B12 (pg/mL)	35	287.0 (240,380)	5 (14.3%)	15	305.0 (146.54)	3 (20.0%)	50	288.0 (235.0,374.0)	8 (16.0%)
Parathyroid Hormone (pg/mL)	34	83.5 (66,111)	15 (44.1%)^c^	15	92.9 (57.87)	7 (46.7%)^b^	49	85.0 (58,111)	22 (44.9%)^c^
Calcium (mg/dL)	35	8.8 (0.39)	3 (8.6%)^d^	17	8.9 (0.5)	1 (5.9%)	52	8.8 (0.43)	4 (7.7%)^d^
Vitamin D (ng/mL)	35	15.2 (8.31)	27 (77.1%)	15	13.7 (8.01)	12 (80.0%)	50	14.8 (8.16)	39 (78.0%)
Alkaline Phosphatase (U/L)	35	89.8 (34.46)	1 (2.9%)^c^	17	86.9 (30.95)	1 (5.9%)^b^	52	88.9 (33.08)	2 (3.9%)^c^
Folate (ng/mL)	32	543.5 (211.76)	0 (0.0%)	15	393 (327,503)	0 (0.0%)	47	470.0 (381.0,597.0)	0 (0.0%)

a The reference ranges used to determine abnormal values are provided in the Supplemental Appendix.

b These columns report the number and proportion of abnormally low values unless otherwise noted.

c For parathyroid hormone and alkaline phosphatase, abnormally high values are reported in these cells

d For calcium, abnormally low values are being reported; there were no instances of high abnormal calcium values

**Table 6.** Clinical Events.

	Subjects, no. (%)	Events, no.	Rate (95% CI)^a^
Obstetric	17 (45.9%)	25	85.9 (58.0, 127.1)
Gynecologic	7 (18.9%)	20	68.7 (44.3, 106.5)
Upper Endoscopy	13 (22.4%)	29	62.4 (43.3, 89.7)
Cholecystectomy	12 (20.7%)	12	25.8 (14.7, 45.4)
Excess skin removal	8 (13.8%)	11	23.7 (13.1, 42.7)
Blood transfusion	2 (3.4%)	3	6.5 (2.1, 20.0)
Colonoscopy	2 (3.4%)	3	6.5 (2.1, 20.0)
Parenteral infusion for micronutrient deficiency	2 (3.4%)	3	6.5 (2.1, 20.0)
Repair GI perforation	3 (5.2%)	3	6.5 (2.1, 20.0)
Appendectomy	2 (3.4%)	2	4.3 (1.1, 17.2)
Exploratory laparoscopy/laparotomy	2 (3.4%)	2	4.3 (1.1, 17.2)

a Events per 1000 person-years (i.e., 100 subjects followed for 10 years).
