# Supplementary material for: Bariatric Surgery Meaningfully and Durably Improves Long-term Outcomes in Adolescents with Severe Obesity
Source: Lancet Diabetes Endocrinol. Author manuscript; Available in PMC 2021 Jul 15. (PMC8282411; doi:10.1016/S2213-8587(16)30315-1)
Supplement: Supplementary Appendix [file NIHMS842448-supplement-Supplementary_Appendix.docx]

**Supplementary webappendix**

This appendix has been provided by the authors to give readers additional information about their work.

Supplement to: Inge TH, Jenkins TM, Xanthakos SA, Dixon JB, Daniels, SR, Zeller MH, Helmrath MA. Bariatric Surgery Meaningfully and Durably Improves Long-term Outcomes in Adolescents with Severe Obesity

**Table of Contents**

**Page**

A. Acknowledgements 3

B. Supplemental methodologic and clarifying details pertaining to deaths 4

C. Supplemental Data 6

D. Clinical care 9

E. References 12

**A. Acknowledgements**

The authors gratefully acknowledge the expertise and assistance of Jennifer Black, MSSA, LSW, Lindsey Shaw, MS, Tawny W. Boyce, MS, MPH, April Carr, BS, Nina Cunning, Faye Doland, BS, Andrea Ferris, BS, Allison Greenberg, BA, Patricia Hirsch, RN, Renee Jeffreys, PhD, Linda Kollar, RN, Rosie Miller, RN, CCRC, Ashley Morgenthal, BS, Dana Raab, RN, Cynthia Spikes, CRC, Jessica Woo, PhD and data processing and data management staff in the Division of Biostatistics and Epidemiology at Cincinnati Children’s Medical Center. In addition, the authors appreciate the insightful and helpful discussions with Drs. Robert Siegel, Shelley Kirk, Aaron Kelly, Larry Dolan, Marc Michalsky, and Anita Courcoulas during preparation of the manuscript.

**B. Supplemental methodologic and clarifying details pertaining to deaths**

**Study design**

The original Follow-up of Adolescent Bariatric Surgery (FABS) study was an observational study that enrolled participants who underwent surgery during 2001-2007 and collected baseline and annual follow-up anthropometric and health data during routine bariatric clinical visits. Declining follow-up for annual bariatric clinic visits over time (i.e., 30% missing annual visits by postoperative year 2 as described[^1^](#_ENREF_1) led to incomplete and missing data in the FABS research database. Indeed, upon review of medical records for this gastric bypass cohort over the study period, we found that the proportion of the cohort returning for annual bariatric clinical visits for postoperative years 5, 6, 7 ,8, 9, and 10 was 11%, 7%, 5%, 3%, 0%, and 0%, respectively (unpublished data). Thus the Follow-up of Adolescent Bariatric Surgery at 5+ years (FABS-5+) was therefore designed specifically to fill the void of outcome data by systematically locating, contacting, and collecting key outcomes on adolescents who participated in original FABS and had undergone RYGB ≥5 years previously. Funding for this investigator-initiated extension of original FABS was provided by Ethicon Endosurgery (Cincinnati, OH). All subjects enrolled into FABS-5+ provided informed written consent and all study procedures were approved by the Cincinnati Children’s Medical Center (CCHMC) Institutional Review Board.

**Follow-up study visits and data collection**

FABS-5+ long-term follow-up study data were gathered by trained clinical research coordinators either in-person at CCHMC or in the participant’s home. For participants who chose to have a home visit, a contractor (Examination Management Services, Inc. [EMSI; www.emsinet.com]) dispatched an examiner familiar with the study protocol and trained in the conduct of study procedures. Study equipment (e.g., Tanita scale, Welch Allen Spot Vital Monitor 4200B) was sent by overnight delivery to the examiner for consistency of measures. This home visit option ensured that inability to travel to CCHMC was no barrier to participation.

This FABS-5+ study consisted of one visit per participant along with phone contact(s) to obtain consent and complete health history information, if necessary. At all study visits, health history including comorbidity inventory and healthcare utilization (e.g., hospitalizations, procedures) were obtained by a structured health interview, available upon request to corresponding author. Height was measured to the closest 1.0 mm in standing position. Weight was measured in light clothing to the nearest 0.1 kg on an electronic scale (Tanita model TBF-310, Tokyo, Japan). Blood pressure was obtained using a Welch Allen Spot Vital Monitor 4200B. Each visit lasted approximately 4 hours and participants were compensated for their time and reasonable travel expenses were reimbursed.

**Comorbidity and remission definitions**

The diagnosis of diabetes mellitus at baseline was established by referring physicians and clinical data corroborating the diagnosis was confirmed by individual chart review by study investigators. Confirmation included review of medication use for diabetes, HbA1c and fasting blood glucose analyses, and prior medical records from referring primary care physician and/or endocrinologist. Due to the fact that metformin can be used for insulin resistance, weight management, and for polycystic ovary syndrome, participants who were on metformin at baseline with no other indication of a prior diagnosis of diabetes and no laboratory findings consistent with the diagnosis of diabetes were not considered to have diabetes. At long-term follow up, diabetes was defined as either taking medications for diabetes, or HbA1c ≥ 6.5%, or if HbA1c was not available, by fasting glucose ≥ 7 mmol/l, or if laboratory values were unavailable, by self-report during the structured health interview. Remission of diabetes was defined for those who met criteria for T2DM at baseline as: HbA1c <5.7 on no T2DM medications at follow-up.

Hypertension (HTN) at baseline and follow-up was defined as systolic blood pressure or diastolic blood pressure ≥ 95th percentile for gender/height or if systolic blood pressure or diastolic blood pressure were ≥140mmHg or ≥90mmHg, respectively, or if medication was used for control of blood pressure. Definition of remission of HTN for those who were hypertensive at baseline: normal blood pressure on no antihypertension medications at follow-up.

Dyslipidemia was defined at baseline and follow-up as having either an elevated low-density lipoprotein cholesterol (LDL) level (≥3.36 mmol/l if age <21 or ≥4.14 mmol/l if age ≥ 21), or depressed high-density lipoprotein cholesterol (HDL) level (<1.03 mmol/l or <1.29 mmol/l for males and females, respectively), or elevated fasting triglyceride level (≥1.47 mmol/l if age <21 or >2.26 mmol/l if age ≥ 21), or if using a lipid-lowering medication. The definition of remission of dyslipidemia was: normal blood lipids on no lipid lowering medications at follow-up.

**Death of a FABS-5+ participant and a non-participant in the reference surgical population**

Late deaths following adolescent bariatric surgery have been previously reported at 2 and 6 years postoperatively[^2^](#_ENREF_2). Similarly, we know of two deaths of patients who underwent surgery during the period of study in our single center experience. These details are being included in this supplementary material and not in the main manuscript for reasons detailed below. The first death of a patient who underwent surgery during the period of study (2001-2007) occurred before the patient was eligible for long-term follow up. This patient was also a male, 18 years old with a BMI of 80 kg/m^2^ at the time of laparoscopic gastric bypass. He did not participate in this long-term follow-up study as he died nine months postoperatively due to complications of C. difficile colitis and this death was described and published previously[^3^](#_ENREF_3). The second death occurred in an individual who did participate in this long-term follow-up study at his postoperative year 6. However, his death was 2 years after completion of his long term study visit, as reported to authors by his parents. This participant was 18 years old with a BMI of 67 kg/m^2^ at the time of gastric bypass. His death, in postoperative year 8, was due to an apparent substance misuse event.

These mortality events, while few in any single center experience, highlight the complexities of medical conditions treated, postoperative risk taking behaviors, and the need for continued clinical follow-up and treatment.

C. **Supplemental data**

Table S1: Relationship between FABS-5 long-term BMI and risk of Dyslipidemia and Hypertension

| Outcome | Predictor | Risk Ratio (RR) | 95% CI | p-value |
| --- | --- | --- | --- | --- |
| Dyslipidemia | BMI (per 10 units) | 1.34 | 1.09, 1.66 | 0.0060 |
| Hypertension | BMI (per 10 units) | 1.46 | 1.10, 1.94 | 0.0084 |

Table S2: Relationship between FABS-5 long-term BMI and multiple cardiometabolic risk factors

| Outcome | Beta (BMI) | 95% CI | p-value |
| --- | --- | --- | --- |
| Insulin (log) | 0.025 | 0.012, 0.038 | 0.0004 |
| Glucose | 0.04 | -0.23, 0.32 | 0.7572 |
| Hs-CRP (log) | 0.0659 | 0.0269, 0.1049 | 0.0015 |
| HOMA-IR (log) | 0.0238 | 0.0085, 0.0392 | 0.0031 |
| HbA1c | -0.002 | -0.012, 0.009 | 0.7823 |
| Triglycerides (log) | 0.013 | 0.001, 0.025 | 0.0319 |
| LDL | 0.47 | -0.23, 1.17 | 0.1845 |
| HDL | -0.55 | -0.97, -0.13 | 0.0107 |
| Systolic blood pressure | 0.22 | -0.10, 0.54 | 0.1796 |
| Diastolic blood pressure | 0.36 | 0.11, 0.60 | 0.0053 |

Table S3: Descriptive characteristics of the surgical patients included and not included in the manuscript analyses.

|  | Participants  (N=58) | Non-Participants  (N=14) | p-value |
| --- | --- | --- | --- |
| Sex, n (%) |  |  | 0.36 |
| Female | 37 (63.8%) | 11 (78.6%) |  |
| Male | 21 (36.2%) | 3 (21.4%) |  |
|  |  |  |  |
| Race, n (%) |  |  | 0.99 |
| White | 50 (86.2%) | 12 (85.7%) |  |
| Non-White | 8 (13.8%) | 2 (14.3%) |  |
|  |  |  |  |
| Age at surgery, mean (SD) | 17.1 (1.71) | 17.0 (1.43) | 0.84 |
|  |  |  |  |
| Baseline BMI, mean (SD) | 58.5 (10.46) | 64.3 (13.71) | 0.08 |

Figure S1: Graphical depiction of relationship between risk of dyslipidemia and BMI at long-term follow-up.


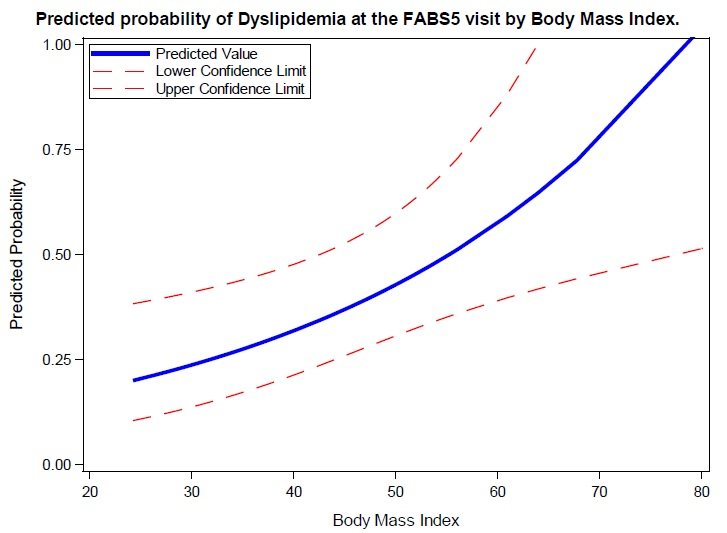


Figure S2: Graphical depiction of relationship between risk of hypertension and BMI at long-term follow-up.

D. Comment regarding clinical care

Our program’s routine clinical micronutrient supplementation during the time period of enrollment (2000-2007) was in line with clinical recommendations at that time which included: 2 multivitamin plus mineral tablets orally per day, to include at least 65 mg elemental iron per day, calcium citrate 1500 mg orally per day, Vitamin D minimum 1000 units orally per day, Vitamin B12 350 mcg sublingual daily and 50 mg thiamine orally daily for first 6 months. We have included this information in the Web Appendix. More recent nutritional guidelines published in 2013 now recommend 3000 units per day of vitamin D and titrating to achieve vitamin D levels >30 ng/mL [^4^](#_ENREF_4).

E. References

1. Jenkins TM, Xanthakos SA, Zeller MH, Barnett SJ, Inge TH. Distance to clinic and follow-up visit compliance in adolescent gastric bypass cohort. *Surg Obes Relat Dis* 2011; **7**(5): 611-5.

2. Sugerman HJ, Sugerman EL, DeMaria EJ, et al. Bariatric surgery for severely obese adolescents. *J Gastrointest Surg* 2003; **7**(1): 102-7; discussion 7-8.

3. Lawson ML, Kirk S, Mitchell T, et al. One-year outcomes of Roux-en-Y gastric bypass for morbidly obese adolescents: a multicenter study from the Pediatric Bariatric Study Group. *Journal of pediatric surgery* 2006; **41**(1): 137-43; discussion -43.

4. Mechanick JI, Youdim A, Jones DB, et al. Clinical practice guidelines for the perioperative nutritional, metabolic, and nonsurgical support of the bariatric surgery patient--2013 update: cosponsored by American Association of Clinical Endocrinologists, The Obesity Society, and American Society for Metabolic & Bariatric Surgery. *Obesity (Silver Spring)* 2013; **21 Suppl 1**: S1-27.
